# Supplementary material for: Modeling glioblastoma heterogeneity as a dynamic network of cell states
Source: Mol Syst Biol. 2021 Sep 16;17(9):e10105. doi: 10.15252/msb.202010105 (PMC8444284; doi:10.15252/msb.202010105)
Supplement: Supplementary file 6 — Source Data for Figure 5 [file MSB-17-e10105-s004.zip › Figure5A_sourcedata/GSEA_3017/hallmarks_stateF.GseaPreranked.1621934628066/HALLMARK_IL2_STAT5_SIGNALING.html]

Details for gene set HALLMARK\_IL2\_STAT5\_SIGNALING[GSEA]

|  || Dataset | state33017 |
| Phenotype | NoPhenotypeAvailable |
| Upregulated in class | na\_pos |
| GeneSet | HALLMARK\_IL2\_STAT5\_SIGNALING |
| Enrichment Score (ES) | 0.4357721 |
| Normalized Enrichment Score (NES) | 2.1135905 |
| Nominal p-value | 0.0 |
| FDR q-value | 0.0020591388 |
| FWER p-Value | 0.016 |
Table: GSEA Results Summary

  

Fig 1: Enrichment plot: HALLMARK\_IL2\_STAT5\_SIGNALING      
 Profile of the Running ES Score & Positions of GeneSet Members on the Rank Ordered List

  

| PROBE | GENE SYMBOL | GENE\_TITLE | RANK IN GENE LIST | RANK METRIC SCORE | RUNNING ES | CORE ENRICHMENT || 1 | HOPX |  |  | 14 | 1.345 | 0.0627 | Yes |
| 2 | APLP1 |  |  | 28 | 1.047 | 0.1106 | Yes |
| 3 | TNFRSF21 |  |  | 36 | 1.016 | 0.1596 | Yes |
| 4 | IFITM3 |  |  | 38 | 0.987 | 0.2098 | Yes |
| 5 | SPP1 |  |  | 57 | 0.898 | 0.2477 | Yes |
| 6 | SERPINB6 |  |  | 83 | 0.791 | 0.2770 | Yes |
| 7 | ANXA4 |  |  | 127 | 0.703 | 0.2937 | Yes |
| 8 | CD44 |  |  | 191 | 0.600 | 0.2961 | Yes |
| 9 | RRAGD |  |  | 218 | 0.578 | 0.3140 | Yes |
| 10 | SLC2A3 |  |  | 228 | 0.569 | 0.3391 | Yes |
| 11 | CA2 |  |  | 243 | 0.558 | 0.3614 | Yes |
| 12 | PRNP |  |  | 269 | 0.538 | 0.3778 | Yes |
| 13 | CSF1 |  |  | 333 | 0.495 | 0.3747 | Yes |
| 14 | ITGAV |  |  | 344 | 0.490 | 0.3954 | Yes |
| 15 | SYT11 |  |  | 352 | 0.486 | 0.4171 | Yes |
| 16 | ADAM19 |  |  | 382 | 0.477 | 0.4285 | Yes |
| 17 | SPRY4 |  |  | 443 | 0.453 | 0.4247 | Yes |
| 18 | IGF2R |  |  | 470 | 0.444 | 0.4358 | Yes |
| 19 | SOCS2 |  |  | 555 | 0.412 | 0.4190 | No |
| 20 | GABARAPL1 |  |  | 654 | 0.384 | 0.3945 | No |
| 21 | RGS16 |  |  | 691 | 0.373 | 0.3974 | No |
| 22 | RORA |  |  | 783 | 0.351 | 0.3744 | No |
| 23 | ARL4A |  |  | 889 | 0.328 | 0.3438 | No |
| 24 | PLEC |  |  | 914 | 0.324 | 0.3496 | No |
| 25 | BHLHE40 |  |  | 928 | 0.321 | 0.3602 | No |
| 26 | AHR |  |  | 942 | 0.319 | 0.3707 | No |
| 27 | CD81 |  |  | 947 | 0.318 | 0.3852 | No |
| 28 | BCL2L1 |  |  | 949 | 0.317 | 0.4011 | No |
| 29 | PLPP1 |  |  | 1017 | 0.307 | 0.3866 | No |
| 30 | IFNGR1 |  |  | 1289 | 0.269 | 0.2781 | No |
| 31 | ITGAE |  |  | 1476 | -0.251 | 0.2071 | No |
| 32 | GSTO1 |  |  | 1525 | -0.265 | 0.1991 | No |
| 33 | PHTF2 |  |  | 1529 | -0.267 | 0.2114 | No |
| 34 | EMP1 |  |  | 1598 | -0.285 | 0.1954 | No |
| 35 | PHLDA1 |  |  | 1718 | -0.324 | 0.1583 | No |
| 36 | PTCH1 |  |  | 1857 | -0.393 | 0.1162 | No |
| 37 | CDC6 |  |  | 1991 | -0.483 | 0.0809 | No |
| 38 | ODC1 |  |  | 2128 | -0.718 | 0.0564 | No |
Table: GSEA details [plain text format]

  

Fig 2: HALLMARK\_IL2\_STAT5\_SIGNALING: Random ES distribution      
 Gene set null distribution of ES for **HALLMARK\_IL2\_STAT5\_SIGNALING**
